# Dietary saponins of sea cucumber alleviate orotic acid-induced fatty liver in rats via PPARα and SREBP-1c signaling

**DOI:** 10.1186/1476-511X-9-25

**Published:** 2010-03-09

**Authors:** Xiao-Qian Hu, Yu-Ming Wang, Jing-Feng Wang, Yong Xue, Zhao-Jie Li, Koji Nagao, Teruyoshi Yanagita, Chang-Hu Xue

**Affiliations:** 1 College of Food Science and Engineering, Ocean University of China, Qingdao, China; 2 Laboratory of Nutrition Biochemistry, Department of Applied Biological Sciences, Saga University, Saga, Japan

## Abstract

**Background:**

Nonalcoholic fatty liver disease is the most common chronic liver disease in the world, and is becoming increasingly prevalent. Saponins of sea cucumber (SSC) are proven to exhibit various biological activities. Therefore, the present study was undertaken to examine the effect of saponins extracted from sea cucumber (Pearsonothuria graeffei) on the preventive activity of fatty liver in rats.

**Methods:**

Male Wistar rats were randomly divided into five groups, including normal control group, fatty liver model group, SSC-treated group with SSC at levels of 0.01%, 0.03% and 0.05%. Model rats were established by administration with 1% orotic acid (OA). After the experiment period, serum total cholesterol (TC), triglyceride (TG), and hepatic lipid concentrations were determined. To search for a possible mechanism, we examined the changes of key enzymes and transcriptional factors involved in hepatic lipids biosynthesis, fatty acid β-oxidation.

**Results:**

Both 0.03% and 0.05% SSC treatment alleviated hepatic steatosis and reduced serum TG and TC concentration significantly in OA fed rats. Hepatic lipogenic enzymes, such as fatty acid synthase (FAS), malic enzyme (ME), and glucose-6-phosphate dehydrogenase (G6PDH) activities were inhibited by SSC treatment. SSC also decreased the gene expression of FAS, ME, G6PDH and sterol-regulatory element binding protein (SREBP-1c). Otherwise, the rats feeding with SSC showed increased carnitine palmitoyl transferase (CPT) activity in the liver. Hepatic peroxisome proliferator-activated receptor (PPARα), together with its target gene CPT and acyl-CoA oxidase (ACO) mRNA expression were also upregulated by SSC.

**Conclusions:**

According to our study, the lipids-lowering effect of dietary SSC may be partly associated with the enhancement of β-oxidation via PPARα activation. In addition, the inhibited SREBP-1c- mediated lipogenesis caused by SSC may also contribute to alleviating fatty liver.

## Background

Nonalcoholic fatty liver disease (NAFLD) evolves when large amounts of lipids deposit in the hepatocytes with no excessive alcohol consumption [1]. Population studies show that NAFLD is strongly associated with the clinical features of the metabolic syndrome such as obesity [2,3], type II diabetes [4], and dislipidemia [5]. The etiological association between NAFLD and metabolic syndrome has been shown in both obese and non-obese patients, and this leads to the suggestion that NAFLD is the liver manifestation of metabolic syndrome [6]. NAFLD is the most common liver disease in western countries, and it is becoming increasingly prevalent in Asian-Pacific regions because of the increasing westernization lifestyle [7]. According to Angulo's review, NAFLD affects 10 to 24 percent of the general population in various countries, and it is increasingly diagnosed in children and adolescents [8]. Therefore more and more researchers focus on the prevention or therapy on NAFLD.

There is growing evidence that dietary sea cucumber could improve lipids metabolism in rats [9,10]. Sea cucumber, is a traditional seafood and important medical material in Asian countries, rich in various bioactive substance. Saponin (also called triterpene glycoside), is the most important secondary metabolite and bioactive composition of sea cucumber, which has been proven to exhibit various biological activities, such as including antifungal, anti-angiogenesis, anti-tumor and immunomodulatory effects [11-13]. However it is unknown whether saponins of sea cucumber (SSC) may improve lipid metabolism in animal models. Several lines of evidence indicate that orotic acid (OA) administration induces fatty liver [14,15]. It may therefore be useful as an animal model for deeper understanding of biochemical changes that occurs during the development of NAFLD.

The aim of this work is to assess the effects of SSC on preventing hepatic lipid accumulation in OA-induced NAFLD rats. To investigate the possible mechanism, we examined the key enzymes activities related in fatty acid biosynthesis and β-oxidation. To gain further insights into the molecule mechanism by which SSC alters gene expression of hepatic lipids metabolism, sterol-regulatory element binding protein (SREBP-1c) mRNA, the principal regulator of hepatic fatty acid biosynthesis, was determined. Meanwhile, the mRNA expression of its response genes, FAS, ME and G6PDH, were also determined. Besides the genes involved in fatty acid de novo synthesis, the genes involved in fatty acid β-oxidation, like PPARα and its target genes, CPT and ACO, were also measured in this study.

## Materials and methods

### Preparation of saponins from sea cucumber

Saponins were isolated from the sea cucumber, *Pearsonothuria graeffei*. Air-dried body walls (500 g) of *Pearsonothuria graeffei *were grinded into powder and extracted four times with refluxing ethanol. The combined extracts were evaporated in vacuo and further partitioned between water and chloroform. The water layer was extracted with n-butanol and the organic layer was evaporated in vacuo to yield n-butanol extracts. The n-butanol extracts were concentrated, and the extracted residue was dissolved in water. Samples were applied with a HP20 resin column, eluted with water, 80% ethanol and 100% ethanol in sequence. The fraction eluted with 80% ethanol was collected and evaporated, which is crude saponin with a final weight of 51.8 g.

### Animals and diets

All aspects of the experiment were conducted according to guidelines provided by the ethical committee of experimental animal care at Ocean University of China (OUC, China).

Male Wistar rats aged 5 wk were purchased from Vital River (Beijing, China), weighting 130-140 g. The rats were housed individually all through the feeding experiment, in a room maintained at a 12 h light/dark cycle, a constant temperature of 24°C and relative humidity of 65 ± 15%. After a 5-day adaptation period, rats were randomly divided into five groups consisting of seven rats each with similar mean body weight (120 ± 2 g). Rats were assigned to the following five groups: 1) a control group (Con) fed AIN-93G diet; 2) a model group (OA) fed AIN-93G diet but containing 1% OA; 3) a group (OA+0.01%) fed the diet containing but containing 1% OA plus 0.01% SSC; 4) a group (OA+0.03%) fed the diet containing but containing 1% OA plus 0.03% SSC; 5) a group (OA+0.05%) fed the diet containing but containing 1% OA plus 0.05% SSC. Experimental diets were prepared according to recommendations of the American Institute of Nutrition (AIN). The diet composition and SSC concentration of the experimental diets were summarized in Table 1. All rats had free access to water and food for 10 days. At the end of the feeding period, rats were killed by bleeding from the abdominal aorta under diethyl ether anesthesia after overnight fasting. Serum was separated from whole blood by centrifugation at 1500 g for 10 min at 4°C. Liver, perirenal adipose and epidymal adipose were quickly excised, washed with ice-cold isotonic saline. After excess water on the surface was removed by blotting with filter paper, the tissues were weighed, frozen in liquid nitrogen and stored at -80°C until analysis.

**Table 1:** Compositions of experimental diets (g/kg diet)

	Con	OA	OA+0.01%	OA+0.03%	OA+0.05%
Casein	200	200	200	200	200
Cornstarch	500	490	489.9	489.7	489.5
Sucrose	100	100	100	100	100
Corn oil	100	100	100	100	100
Mineral mix	35	35	35	35	35
Vitamin mix	10	10	10	10	10
Choline bitartrate	2	2	2	2	2
Cellulose	50	50	50	50	50
DL-Methionine	3	3	3	3	3
OA	--	10	10	10	10
SSC	--	--	0.1	0.3	0.5

Vitamin mixture (AIN-93VX; Dyets Inc.)

Mineral mixture (AIN-93G; Dyets Inc.)

### Serum and liver lipids determination

Serum TG and TC concentrations were determined using enzymatic reagent kits from Biosino (Beijing, China) according to the manufacturer's instructions. Hepatic lipids were extracted with chloroform-methanol 2:1 as Folch *et al*. described [16], and then dissolved using Triton X-100. TG and TC concentrations in liver were determined using enzymatic reagent kits (Biosino, China), and hepatic phospholipids levels were measured by the methods of Bartlett [17].

### Preparation of liver subcellular fractions

A piece of liver was homogenized in 6 vol of a 0.25-M sucrose solution containing 1 mM of EDTA in 10 mM of Tris-HCl buffer (pH 7.4). After precipitating the nuclei fraction, the supernatant was centrifuged at 10 000 g for 10 min at 4°C to obtain mitochondria. The resulting supernatant was recentrifuged at 125 000 g for 60 min to precipitate microsomal, and the remaining supernatant was used as the cytosol fraction. The microsomal pellet was resuspended in a 0.25 M of sucrose solution as described above. Protein concentration was determined by the method of Lowry *et al*. [18], with bovine serum albumin used as the standard.

### Assays of hepatic enzyme activity

The enzyme activity of fatty acid synthase (FAS; EC2.3.1.85) was determined according to the procedure described by Ikeda [19]. The reaction solution contained 0.1 M of phosphate buffer (pH 7.0), 0.2 M of EDTA, 50 μM of acetyl coenzyme A (CoA) and 0.3 mM of reduced nicotinamide adenine dinucleotide phosphate. The entire solution was equilibrated at 30°C. The reaction was initiated by the addition of 0.8 mL cytosol fraction and absorbance was monitored for 2 min at 340 nm. The malonyl-CoA-dependent rate was monitored for 3 min.

The enzyme activity of malic enzyme (ME; EC1.1.1.40) was determined as Ochoa described previously [20]. The reaction solution contained 64 mM of triethanolamine hydrochloride (pH 7.4), 1.2 mM of malic acid, 1.2 mM of oxidized nicotinamide adenine dinucleotide phosphate, and 4 mM of MnCl2. The reaction was initiated by the addition of 0.6 to 0.8 mg of a protein source (cytosol) in a final assay volume of 1 mL at 27°C and absorbance was monitored at 340 nm for 2 min.

The enzyme activity of glucose-6-phosphate dehydrogenase (G6PDH; EC1.1.1.49) was assayed using the method of Glock [21]. The reaction solution contained 0.16 M of Tris-HCl buffer (pH 7.6), 30 mM of MgCl2, 3.3 mM of glucose-6-phosphate, 1.6 mM of oxidized nicotinamide adenine dinucleotide phosphate, and 1 U of 6-phosphogluconate dehydrogenase. The reaction was initiated by the addition of 0.3 to 0.4 mg of a protein source (cytosol) in a final assay volume of 1 mL at 30°C and the absorbance was monitored at 340 nm for 2 min.

The enzyme activity of carnitine palmitoyl transferase (CPT; EC2.3.1.23) was determined as described previously [19]. The reaction solution contained 58 mM of Tris-HCl buffer (pH 8.0), 1.25 mM of EDTA, 1.25 mM of L-carnitine, 0.25 mM of 5,5'-dithiobis-2-nitrobenzoic acid, 37.5 μM of palmitoyl-CoA, and 0.1% Triton-X. The entire solution was equilibrated at 27°C. The reaction was initiated by the addition of 0.2 mL mitochondri fraction and absorbance was monitored for 5 min at 412 nm. The L-carnitine-independent rate was monitored for 5 min. The difference between with and without L-carnitine gave the L-carnitine-dependent rate for formation of CoA-SH.

### Analysis of mRNA expression of hepatic genes

For analysis of gene expression, total RNA was extracted from 100 mg frozen liver samples using Trizol reagent (Invitrogen, USA). RNA was quantified by A260 and its integrity verified by agarose gel electrophoresis using ethidium bromide for visualization. 1 μg total RNA and random primer (TOYOBO, Japan) were used for cDNA synthesis.

The concentration of cDNA was analyzed by real-time detection PCR (ABI Prism 7500 Sequence Detection System, USA) using Sybr Green I Master Mix (TOYOBO, Japan). PCR was carried out with a final volume of 30 μL reaction mixture containing: 15 μL master mix, 2 μL first-strand cDNA, 1 μL primer with each forward and reverse, and H_2_O to make up 30 μL. A dilution curve from one cDNA source using dilutions of 1:2, 1:4, 1:8 and a no-template control was run for each gene. The gene expression was determined by relative quantification using the standard curve method. A final melting curve guaranteed the authenticity of the target product. The expression signal of the housekeeping gene β-actin served as an internal control for normalization.

The primer sequences used for real-time PCR were as follows (Table 2):

**Table 2:** Primers used in this study

Gene	Forward/reverse primer	**Accession No**.	Product length
β-actin	GCAGATGTGGATCAGCAAGC GTCAAAGAAAGGGTGTAAAACG	NM_031144	111 bp
FAS	GGAACTGAACGGCATTACTCG CATGCCGTTATCAACTTGTCC	X62888	153 bp
ME	TCACCTGCCCTAATGTCCCT CATGCCGTTATCAACTTGTCC	NM_012600	185 bp
G6PDH	GTTTGGCAGCGGCAACTAA GGCATCACCCTGGTACAACTC	NM_017006	108 bp
CPT-1	GCTTCCCCTTACTGGTTCC AACTGGCAGGCAATGAGACT	NM_031559	115 bp
CPT-2	GCCCAAACCCCATTTTCTA TAGGCAGAGGCAGAAGACAGCA	NM_012930	186 bp
ACO	ACTATATTTGGCCAATTTTGTG TGTGGCAGTGGTTTCCAAGCC	NM_017340	154 bp
SREBP-1c	CGCTACCGTTCCTCTATCAA TTCGCAGGGTCAGGTTCTC	AF286470	166 bp
PPARα	GAAGCAGATGACCTGGAAAGT AGCCTGGACAGCTCCCTAA	NM_013196	144 bp

Gene-specific primers were designed by Primer Premier 5.0 software.

### Statistical analyses

All statistical analyses were performed using PC SAS software. One-way ANOVA and Tukey's post hoc test with least significant difference were used to compare group means. Homogeneity of variances was tested by Levene's test and Welch's ANOVA was used to compare group means when the group variances were unequal. All the values in tables and figures are expressed as mean ± standard error of the mean. *P *< 0.05 was considered statistically significant.

## Results

### General observations

The initial body weight was similar in five groups (data not shown). After 10 days experiment, there is no difference in daily food intake among any group. OA feeding did not affect body weight, whereas the body weight was slightly decreased by dietary SSC. The rats feed with OA resulted in a marked decrease in epididymal adipose weight (*P *< 0.05) and had no change in perirenal adipose tissue weight. Whereas 0.05% SSC treatment significantly reduced perirenal adipose weight in OA-fed rats (*P *< 0.05). OA treatment significantly increased liver weight compared with the control group (*P *< 0.01), indicating lipids accumulation in the liver. Whereas SSC did not affect liver weight in the OA-fed rats (Table 3).

**Table 3:** The effect of SSC on body weight, food intake, adipose weight and liver weight in rats

	Con	OA	OA+0.01%	OA+0.03%	OA+0.05%
Body weight, *g*	198.33 ± 6.76	199.71 ± 5.53	195.15 ± 4.74	196.00 ± 6.24	193.00 ± 7.93
Food intake, *g/d*	21.42 ± 0.90	20.77 ± 1.07	21.21 ± 0.93	21.81 ± 1.01	21.19 ± 1.05
Perirenal adipose, *g/100 g BW*	0.31 ± 0.04	0.34 ± 0.02	0.32 ± 0.03	0.29 ± 0.04	0.24 ± 0.03*
Epididymal adipose, *g/100 g BW*	0.67 ± 0.04	0.78 ± 0.03^#^	0.82 ± 0.04	0.78 ± 0.05	0.73 ± 0.06
Liver weight, *g/100 g BW*	3.83 ± 0.05	4.37 ± 0.18^##^	4.39 ± 0.11	4.28 ± 0.07	4.39 ± 0.11

Data were given as mean ± standard error of the mean of seven rats. # P < 0.05, # # P < 0.01 different from the control group; * P < 0.05 different from the OA-fed group.

Rats were fed experimental diets for 10 d.

### Serum and hepatic lipids levels

Compared with control group, administration of OA increased serum TG (*P *< 0.01) and TC concentration. All dose SSC treatment groups tended to have lower serum lipids than that of OA group, both 0.03% and 0.05% SSC showed significant lipid lowering effects on both serum TG (*P *< 0.05, *P *< 0.01) and TC (*P *< 0.01, *P *< 0.01) (Table 4).

**Table 4:** The effect of SSC on serum and hepatic lipids in rats

	Con	OA	OA+0.01%	OA+0.03%	OA+0.05%
Serum lipids					
TC, *mmol/L*	1.98 ± 0.09	2.15 ± 0.10	1.88 ± 0.12	1.64 ± 0.11**	1.57 ± 0.14**
TG, *mmol/L*	0.39 ± 0.01	0.57 ± 0.06^##^	0.47 ± 0.03	0.42 ± 0.05*	0.36 ± 0.02**
Hepatic lipids					
TC, *mg/g*	3.22 ± 0.12	5.50 ± 0.55^##^	4.37 ± 0.49	3.99 ± 0.36**	4.14 ± 0.34*
TG, *mg/g*	6.16 ± 0.65	43.97 ± 3.19^##^	30.11 ± 5.47*	17.02 ± 4.40**	18.73 ± 4.79**
PL, *mg/g*	31.20 ± 0.67	30.13 ± 0.43	28.99 ± 0.73	28.99 ± 0.73	29.51 ± 0.73

Data were given as mean ± standard error of the mean of seven rats. ^# ^P < 0.05, ^## ^P < 0.01 different from the control group; * P < 0.05, **P < 0.05 different from the OA-fed group.

Rats were fed experimental diets for 10 d.

Rats fed the OA diet had a hepatic TG concentration about seven times higher than that in control group (*P *< 0.01). However, dietary SSC attenuated liver TG accumulation in all dose treatment compared with the OA group (*P *< 0.05, *P *< 0.01, *P *< 0.01). In addition, the same trend of hepatic TC concentration was observed with TG between control group and OA group (*P *< 0.01); dietary SSC at 0.03% and 0.05% reduced hepatic TC content (*P *< 0.01, *P *< 0.05). Whereas, there was no difference in hepatic phospholipids content among these groups (Table 4).

### Hepatic enzyme activities involved in lipids biosynthesis

**Figure 1:**
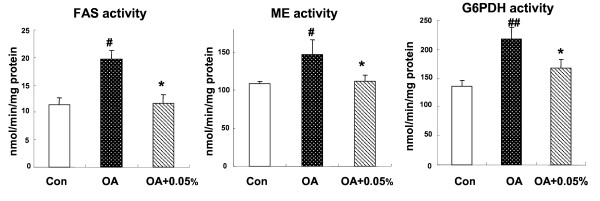
**Effects of SSC on hepatic enzymes activities involved in fatty acid biosynthesis in rats**. The hepatic enzymes activities of FAS, ME and G6PDH in rats fed Con or OA diet or the diet with OA+0.05% SSC for 10 d. Data were given as mean ± standard error of the mean of seven rats. ^# ^P < 0.05, ^## ^P < 0.01 different from the control group; * P < 0.05 different from the OA-fed group.

We examined the activities of critical enzymes in the liver involved in lipids biosynthesis, including FAS, ME and G6PDH. Considering the integrated lipids lowering effects of SSC, we chose 0.05% SSC treated group to access the changes of those enzymes because it exhibited most effective actions. After fed with OA diets for 10 days, the rats showed a significantly increased activity of enzymes all above compared with rats fed normal AIN-93 diet (*P *< 0.01, *P *< 0.05, *P *< 0.05), implying an enhancement of lipogenesis in liver. When giving 0.05% SSC to NAFLD rats, hepatic FAS, ME and G6PDH activities tended to normal level, with a reduction of 41.2% (*P *< 0.01), 23.8% (*P *< 0.05) and 23.0% (*P *< 0.05) separately compared with OA rats (Figure 1).

### Hepatic enzyme activities involved in fatty acid β-oxidation

**Figure 2:**
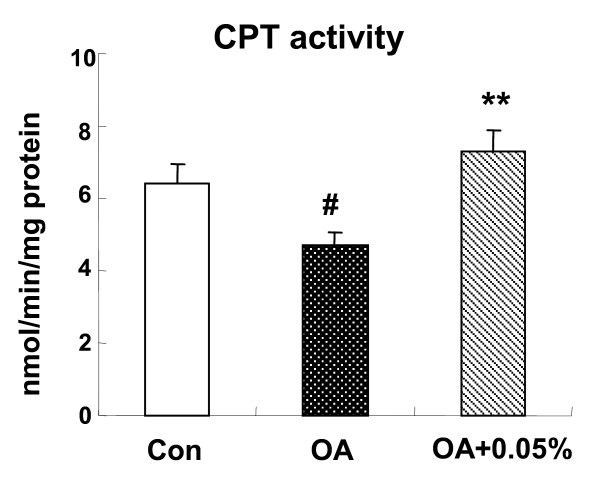
**Effects of SSC on hepatic CPT activity in rats**. The hepatic CPT activity in rats fed Con or OA diet or the diet with OA+0.05% SSC for 10 d. Data were given as mean ± standard error of the mean of seven rats. ^# ^P < 0.05 different from the control group; **P < 0.01 different from the OA-fed group.

The mitochondrial fatty acid β-oxidation in liver was suppressed after OA administration (*P *< 0.05), as measured using hepatic CPT activity. Furthermore, a significant increased CPT activity was observed in OA+0.05% SSC group (*P *< 0.01), even higher than that of control group (Figure 2).

### Relative mRNA concentrations of SREBP-1c and its target genesin the liver

**Figure 3:**
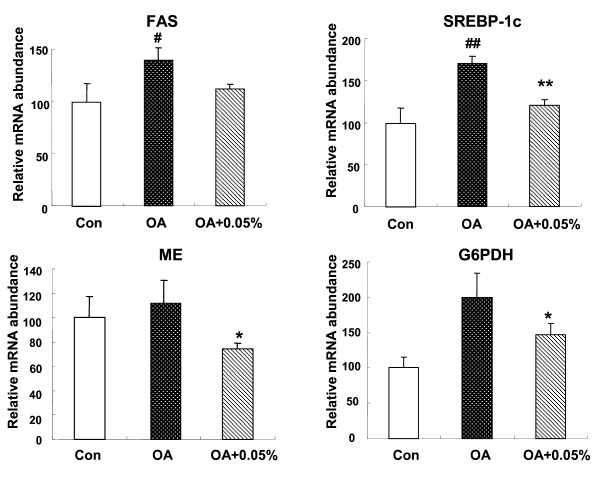
**Effects of SSC on hepatic mRNA expression of lipogenic genes in rats**. The expression of hepatic SREBP-1C, FAS, ME and G6PDH in rats fed Con or OA diet or the diet with OA+0.05% SSC for 10 d. Data were given as mean ± standard error of the mean of seven rats. ^# ^P < 0.05, ^## ^P < 0.01 different from the control group; * P < 0.05, **P < 0.05 different from the OA-fed group.

To explore the molecular mechanism by which SSC may regulate the different transcription factors and enzymes related in lipids metabolism, we examined the expression of hepatic genes that regulate lipogenesis. 1% OA administration markedly increased the expression of hepatic SREBP-1c mRNA (*P *< 0.01) and also the target lipogenic gene. OA caused a 1.4-fold induction of FAS mRNA (*P *< 0.05), and a 2.0-fold induction of G6PDH (*P *< 0.05). Meanwhile, OA increased ME expression but with no significant difference compared with control group. SSC at 0.05% prevented the OA-induced stimulation in SREBP-1c (*P *< 0.01) together with its response gene. FAS, ME and G6PDH mRNA expression was downregulated by SSC treatment, with a decrease of 20.1% (*P *< 0.05), 33.9% (*P *< 0.05) and 26.7% (*P *< 0.05) separately (Figure 3).

### Relative mRNA concentrations of PPARα and its target genes inthe liver

**Figure 4:**
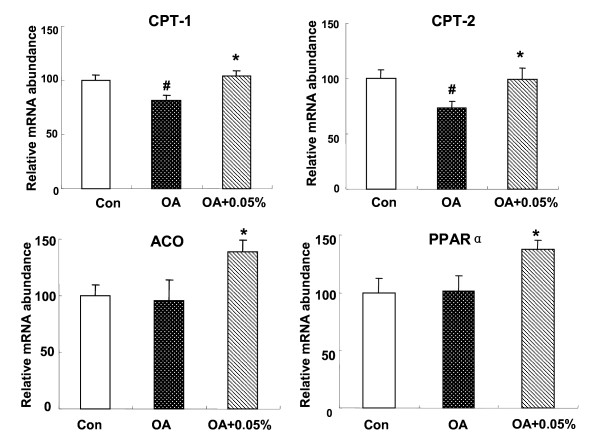
**Effects of SSC on hepatic mRNA expression of lipolytic genes in rats**. The expression of hepatic PPARα, CPT-1, CPT-2 and ACO in rats fed Con or OA diet or the diet with OA+0.05% SSC for 10 d. Data were given as mean ± standard error of the mean of seven rats. ^# ^P < 0.05 different from the control group; * P < 0.05, different from the OA-fed group.

Nonetheless, the reduction of hepatic lipids by SSC could also be a result of an increase mRNA expression in fatty acid β-oxidation. Therefore, we investigated the expression of PPARα and its target gene, CPT-1, CPT-2 and ACO in the liver. The PPARα mRNA level was unchanged between C and OA group, however 0.05% SSC could enhance PPARα mRNA level by 1.37-fold (*P *< 0.05). CPT-1 and CPT-2 showed the same pattern. CPT-1and CPT-2 expression was inhibited by OA administration (*P *< 0.05), and both was stimulated with 0.05% dietary SSC treatment (*P *< 0.05). ACO, a critical enzyme required for peroxisomal β-oxidation, its expression showed no significant difference in control and OA group, whereas it was much higher in SSC-treated group (45.3%, *P *< 0.05) (Figure 4).

## Discussion

It is clearly established that OA administration induces fatty liver. Previous studies have demonstrated that hepatic TG accumulation in rats fed OA as a result of stimulated lipogensis [22,23] and impaired fatty acid catabolism [24]. As we know, the liver plays a central role in lipids metabolism and likely contribute to the onset and progression of several chronic diseases, including atherosclerosis, diabetes, and obesity. Because of the high increasing prevalence of NAFLD in the world, it is important to discover nutrients that will prevent the development of NAFLD.

In the present study, the results showed that 1% OA-supplement diet induced fatty liver in rats as previous studies reported, with a concomitant significant increase in serum TG concentration. And more excitedly, we found that SSC could markedly reduced hepatic lipids accumulation in the fatty liver rats, as well as serum TG and TC concentration. The rats exhibited slight decrease tendency in both serum TG and TC, even if fed at 0.01% SSC; when added 0.03% and 0.05% SSC, the rats had much lower lipids level in serum. It is suggested that dietary SSC could reduce serum lipids in a dose-response manner.

The primary metabolic cause of lipid accumulation in the liver is not well understood, but it could potentially result from alterations in lipogenesis. Some key enzymes involved in fatty acid biosynthesis were measured in this study. FAS, a critical enzyme required for fatty acid de novo synthesis, showed an increased activity in rats feed OA diet. In addition, the activities of ME and G6PDH, which provide NADPH required for FAS, were accelerated by OA administration. The finding is consistent with the report of Buang et al. [23], in which the lipogenic enzymes activities were higher. The present study demonstrated that SSC suppressed all of those lipogenic enzymes activities, suggesting that the inhibition of hepatic endogenous fatty acid synthesis may partly explain why SSC alleviated fatty liver.

SREBP-1c is a member of the family of SREBP membrane-bound transcription factors. It activates mainly the transcription of lipogenic genes that contain sterol-regulatory elements in their promoter regions, such as FAS, G6PHD, ME [25,26]. SREBP-1c plays a major role in the pathogenesis of NAFLD, as suggested in many studies. Overexpression of SREBP-1c produces a pronounced elevation of hepatic TG concentrations leading to the development of NAFLD [27-29]. Hence, it is suggested to suppress SREBP-1c expression in liver may reduce the TG accumulation. Shrestha et al. showed that dietary catechins extracted from green tea extract could reduce plasma and hepatic TG concentration in fructose-fed rats, mainly through downregulation of SREBP-1c and its target gene [30]. Interestingly, we found that the SREBP-1c mRNA expression was stimulated by OA treatment, and consumption of SSC suppressed its expression. As a result of the low SREBP-1c expression in rats fed SSC, there was a concomitant significant reduction in the expression of FAS, ME and G6PDH mRNA. The results suggested that the stimulated FAS, ME and G6PDH transcription may occur after SREBP-1c activation caused by OA. SSC is likely to have direct inhibitory effect on the SREBP-1c expression, which in turn influences these lipogenic genes transcriptions, thereby reduces enzymes activity, resulting in a low rate of lipid synthesis.

Nonetheless, the accumulation of hepatic lipids by OA could also be the result of a decrease in fatty acid β-oxidation. Fatty acid β-oxidation takes place in two cellular organelles: mitochondria and peroxisome [31]. It should be emphasized that mitochondria is the quantitatively dominating organelle in liver cells, proposing that mitochondrial oxidation might have the largest impact on the total β-oxidation [32]. Since CPT is a rate-limiting enzyme of mitochondrial β-oxidation, we examined its activity to reflect β-oxidation activity. And the results showed that OA group rats decreased the β-oxidation capacity of the liver, in agreement with the report of Miyazawa [24]. Dietary SSC increased the hepatic CPT activity by approximately 60%, suggesting an enhancement of β-oxidation. This may contribute to a reduction of hepatic TG deposit and steatosis, because of the reduced flux of fatty acid used for TG synthesis. It is proposed to be one of the mechanisms associated with hypolipidemic effect of SSC.

Since SSC could enhance fatty acid β-oxidation in the liver, we evaluated mRNA expression related to β-oxidation. CPT-1 and CPT-2 were chosen as markers of mitochondria, because they are rate-limiting enzymes of fatty acid β-oxidation and used to reflect mitochondria oxidation activity. ACO was chosen as marker because it is a point to control peroxisomal β-oxidation. As we know, the β-oxidation of lipids in liver could be regulated by different transcription factors. PPARα is thought to be the principal regulator in the fatty acid oxidation. It is highly expressed in tissues obtaining a high level of fatty acid catabolism, such as liver, brown fat, heart, and skeletal muscle [33-35]. PPARα agonists, such as fibrates, have been used for many years for treating dyslipidemia, mainly due to their actions of lowering TG levels [36]. Recently, Huang et al. reported that *Salacia oblonga *extract actions as a PPARα activator to improve hepatic steatosis in obese Zucker rats [37]. To explore whether the hypolipidemic effect of SSC was related to PPARα activation, mRNA expression of PPARα and its response gene were measured using realtime PCR. In the present study, PPARα mRNA expression is significantly up-regulated by SSC treatment, as well as CPT-1, CPT-2 and ACO mRNA. The same trend among these genes suggested that SSC enhanced fatty acid β-oxidation in liver via the pathway of PPARα-mediated gene transcription. The upregulation of gene expression leads to the increased β-oxidation activity.

## Conclusions

In summary, this study, using an OA-induced fatty liver model, provides evidence that SSC significantly alleviated hepatic steatosis and reduced serum TG level. Evidence from animal studies indicates that saponins extracted from a variety of plant sources have lipids lowering effects, such as ginseng, green tea and so on [38-41]. It should be pointed that, saponins used in the current study were prepared from sea cucumber, belongs to the Echinodermata phylum. To our knowledge, this is the first report on the hypolipidmic effect of saponins derived from marine animals. Our findings demonstrated that improvement of fatty liver by SSC may be through the enhancement of PPARα-mediated β-oxidation. On the other hand, reduction of hepatic lipid accumulation was associated with a SREBP-1c- mediated lipogenesis pathway.

## Abbreviations

NAFLD: nonalcoholic fatty liver disease; SSC: saponins of sea cucumber; TG: triglyceride; TC: total cholesterol; OA: orotic acid; FAS: fatty acid synthase; ME: malic enzyme; G6PDH: glucose-6-phosphate dehydrogenase; SREBP: sterol-regulatory element binding protein; CPT: carnitine palmitoyl transferase; PPAR: peroxisome proliferator-activated receptor; ACO: acyl-CoA oxidase.

## Competing interests

The authors declare that they have no competing interests.

## Authors' contributions

XQH and YMW made substantial contributions to the conception and design of the study, performing the experiment, assembly, analysis and interpretation of data and drafting the manuscript. JFW, YX and ZJL participated in experimental work and collection, assembly, analysis of data. KN and TY made contributions to interpretation of data and revision of the manuscript. CHX made contributions to conception and design, analysis and interpretation of data and coordination to draft the manuscript. All authors have read and approved this manuscript.
